# Exploring potential of copper and silver nano particles to establish efficient callogenesis and regeneration system for wheat (*Triticum aestivum* L.)

**DOI:** 10.1080/21645698.2021.1917975

**Published:** 2021-05-03

**Authors:** Waqar Afzal Malik, Imran Mahmood, Abdul Razzaq, Maria Afzal, Ghulam Abbas Shah, Asif Iqbal, Muhammad Zain, Allah Ditta, Saeed Ahmed Asad, Ishfaq Ahmad, Naimatullah Mangi, Wuwei Ye

**Affiliations:** a Institute of Cotton Research of Chinese Academy of Agricultural Sciences / Research Base, Zhengzhou University, State Key Laboratory of Cotton Biology / Key Laboratory for Cotton Genetic Improvement, MOA, Anyang, Henan, China; b Department of Agronomy, PMAS Arid Agriculture University, Rawalpindi, Pakistan; c Department of Botany, University of Gujrat, Gujrat, Pakistan; d Department of agronomy, Farmland Irrigation Research Institute of CAAS, Xinxiang, Henan, China; e Department of Plant Breeding and Genetics, Nuclear Institute for Agriculture and Biology, Faisalabad, Pakistan; f Centre for Climate Research and Development, COMSATS University, Islamabad, Pakistan; g Climate Resilience Department, Asian Disaster Preparedness Center (ADPC), Islamabad, Pakistan

**Keywords:** Nano particles, nanotechnology, callus, tissue culture, mature embryo, explant, cytokinins, auxins

## Abstract

*In vitro* recalcitrance of wheat to regeneration is the major bottleneck for its improvement through callus-based genetic transformation. Nanotechnology is one of the most dynamic areas of research, which can transform agriculture and biotechnology to ensure food security on sustainable basis. Present study was designed to investigate effects of CuSO_4_, AgNO_3_ and their nanoparticles on tissue culture responses of mature embryo culture of wheat genotypes (AS-2002 and Wafaq-2001). Initially, MS-based callus induction and regeneration medium were optimized for both genotypes using various concentrations of auxin (2,4-D, IAA) and cytokinins (BAP, kinetin). The genotypes differed for embryogenic callus induction and regeneration potential. Genotype AS-2002 yielded maximum embryogenic calli in response to 3.0 mg/l 2,4-D, whereas Wafaq-2001 offered the highest embryogenic calli against 3.5 mg/l 2,4-D supplemented in the induction medium. Genotype AS-2002 showed maximum regeneration (59.33%) in response to regeneration protocol comprising 0.5 mg/l IAA, 0.3 mg/l BAP and 1.0 mg/l Kin, while Wafaq-2001 performed best in response to 0.5 mg/l IAA, 0.3 mg/l BAP and 1.5 mg/l Kin with 55.33% regeneration efficiency. The same optimized basal induction and regeneration medium for both genotypes were further used to study effects of CuSO_4_, AgNO_3_ and their nano-particles employing independent experiments. The optimized induction medium fortified with various concentrations of CuSO_4_ or CuNPs confirmed significant effects on frequency of embryogenic callus. Addition of either 0.020 mg/l or 0.025 mg/l CuSO_4_, or 0.015 mg/l CNPs showed comparable results for embryogenic callus induction and were statistically at par with embryogenic callus induction of 74.00%, 75.67% and 76.83%, respectively. Significantly higher regeneration was achieved from MS-based regeneration medium supplemented with 0.015 mg/l or 0.020 mg/l CuNPs than standard 0.025 mg/l CuSO_4_. In another study, the basal induction and regeneration medium were fortified with AgNO_3_ or AgNPs ranging from 1 to 7 mg/l along with basal regeneration media devoid of AgNO_3_ or AgNPs (control). The maximum embryogenic calli were witnessed from medium fortified with 3.0 mg/l or 4.0 mg/l AgNPs compared with control and rest of the treatments. The standardized regeneration medium fortified with 5.0 mg/l AgNO_3_ or 3.0 mg/l AgNPs showed pronounced effect on regeneration of wheat genotypes and offered maximum regeneration compared with control. The individual and combined effect of Cu and Ag nanoparticles along with control (basal regeneration media of each genotype) was also tested. Surprisingly, co-application of metallic NPs showed a significant increase in embryogenic callus formation of genotypes. Induction medium supplemented with 0.015 mg/l CuNPs + 4.0 mg/l AgNPs or 0.020 mg/l CuNPs + 2.0 mg/l AgNPs showed splendid results compared to control and other combination of Cu and Ag nanoparticles. The maximum regeneration was achieved by co-application of 0.015 mg/l CuNP and 4.0 mg/l AgNPs with 21% increment of regeneration over control. It is revealed that CuNPs and AgNPs are potential candidate to augment somatic embryogenesis and regeneration of mature embryo explants of wheat.

**Abbreviations**: 2,4-D (2,4-dichlorophenoxyacetic acid), BAP (6-benzylaminopurine), IAA (Indole-3-acetic acid), AgNPs (silver nanoparticles), CuNPs (copper nanoparticles)

## 1. Introduction

Wheat is the leading cereal food crop in the world, eaten by 2.5 billion people in 89 countries. By 2050, demand for wheat in the developing world is projected to increase by 60%.^1^ Globally, about 2 billion people (26.4%) are facing food insecurity,^2^ which demands a considerable yield boost of wheat. The achievements of desired goals of yield boost seem impracticable by conventional breeding approaches being slow, less selective, restricted gene pool availability and species barrier in addition to other biological constraints. However, exploitation and integration of novel technological means like nanotechnology and recombinant DNA technology can bridge up the yield gap to feed increasing world population.

Until now, genetic transformation of wheat is carried out through agrobacterium-mediated transformation and gene gun involving callus phase. Wherein, major bottleneck is not delivery of the genes, but the establishment of long-term cultures and finally regeneration of transformed cell lines.^3–5^ Media composition, cytokinins to auxins ratio, genotypes, explants and *in vitro* culture conditions are the major restraints for efficient callusing and regeneration of wheat.^6–9^ Development of efficient callus induction and regeneration systems besides selection of tissue culture responsive genotypes are therefore prerequisite for improvement of wheat through modern biotechnological tools.^9–11^ *In vitro* callus culture and regeneration are very much dependent on interactions of natural endogenous growth regulators, genotypes, explants and culture conditions^7,12,13^ . The rate and growth pattern of the explants is influenced by changes in relative concentrations of auxins and cytokinins for various genotypes of wheat. It is likely that more than one combination of two growth regulators or changes in their relative concentration in the tissue culture media may yield optimum results.^8,14,15^

Efforts are being made to augment tissue culture responses of wheat by modifying constituents of tissue culture media especially CuSO_4_ and AgNO_3_. The CuSO_4_ (0.025 mg/l) is an integral component of MS medium,^16^ but copper in its bulk form is vastly utilized as nutritive plant medium in callus induction and regeneration protocols. Cu^2+^ is known to be a cofactor of many important enzymes associated with many biological processes^17^ and plays an important role in plant tissue culture.^18^ Anti-ethylene compounds AgNO_3_ is supplemented in the medium to augment callogenesis and regeneration of plants. The lucrative effects of AgNO_3_ on somatic embryogenesis and regeneration of plants are elucidated in many reports.^19–24^ For example, addition of 10 mg/l AgNO_3_ in MS-based induction medium significantly improved embryogenic callus frequency and callus growth in immature embryo culture of wheat.^24^ Shah *et al.*,^25^ observed an improvement in callus induction frequency of tomato by supplementing 10–15 mg/l AgNO_3_ with 2.0 mg/l IAA and 2.5 mg/l BAP in MS media, while 8–10 mg/l AgNO_3_ with 0.1 mg/l IAA, 1.0 mg/l zeatin and 2.0 mg/l BAP yielded maximum regenerants. Pretreatment of another explant of tomato with 5 mg/l AgNO_3_ yielded maximum callus, shoot induction percentage and plant regeneration.^22^ Similarly, regeneration of Zinnia genotypes was increased when MS medium was fortified with AgNO_3_.^26^ In rice, high somatic embryogenesis of mature embryo explants was achieved from MS medium containing 2 mg/l 2,4-D and 3 mg/l AgNO_3_, while frequency of plant regeneration was higher on medium supplemented with 5 mg/l AgNO_3._^21^ Alike to AgNO_3_, addition of CuSO_4_ along with 2,4-D and zeatin in regeneration medium had also shown promising results for *in vitro* multiple shoot induction in wheat.^14^ For example, 0.1–100 μM CuSO_4_ significantly enhanced shoot regeneration from calli of wheat and triticale, and of tobacco leaf disc cultures.^18^ Immature embryos of indica rice (*Oryza sativa* L.) showed improved somatic embryogenesis on MS medium containing 9.0 μmol/l 2,4-D along with 10.0 or 50.0 μmol/l CuSO_4_. Maximum plants were regenerated on MS medium containing 10.0–50.0 μmol/l CuSO_4_ for various genotypes.^27^ The prediction models suggested that addition of 13.08 mg/l CuSO_4_ in the regeneration medium could significantly improve regeneration potential of immature embryo explant of wheat.^12^

In modern material sciences, nanoparticles (NPs) had gained substantial attention of the researchers. Nanoparticles (NPs) are two-dimensional materials with approximate size of 1–100 nm. The metal NPs had novel properties not exhibited by particles of macro size of the same substance.^14^ Nanoparticles have wide applications in industries and agriculture due to their small size, unique structure and physiochemical properties.^28^ Metal NPs can potentially affect morphological and physiological responses of plants. Improvement in root growth, seed germination, metabolism,^29,30^ photosynthesis, nutrient use efficiency, grain quality and yield had been reported by exposure of seeds and plants with metal NPs.^31^ TiO_2_ nano particles were found effective to augment regeneration of rice callus lines.^32^ Similarly, somatic embryogenesis of banana was increased by supplementing Zn nanoparticles in the media.^33^ Some toxic eﬀects of NPs had been reported on plants and animals, but no reports are available which showed the harmful eﬀects of NPs on tissue culture of plants.^34^

Among metal nanoparticles, NPs of copper and silver had gained immense popularity in life sciences^35^ and biotechnology due to their unique anti-microbial activities.^36^ AgNPs exhibit wide range of antimicrobial^37^ and exceptional antioxidant activity^38–40^ in addition to functional stability, ease of application, heat resistance, nontoxic, environment friendly, cost-effective and easy accessibility.^35^ Application of silver nanoparticles (AgNPs) in plant tissue culture had proved their positive role in callus induction, somatic embryogenesis, organogenesis, somaclonal variation, genetic transformation, and secondary metabolites production.^41^ Supplementing Ag in modified culture media in the form of AgNO_3_ and Ag_2_S_2_O_3_ can improve regeneration of plants. The improvement in regeneration of shoots is more potent when AgNPs are supplemented in the media than its other ionic counterparts, i.e., AgNO_3_ and Ag_2_S_2_O_3_.^42^ Addition of AgNPs in induction medium increases callus growth along with improved antioxidant (catalase, superoxide dismutase and peroxidase) activities.^40^ For instance, callus induction, callus proliferation and regeneration of *Tecomella undulata* were increased in response to fortification of media with AgNPs.^43^ Similarly, exposure of seeds or plants to appropriate concentration of AgNPs had augmented nutrient use efficiency,^38,44^ seed germination, fresh weight, dry weight, chlorophyll contents and grain yield of wheat.^39^ Exogenous application of AgNPs (40 ppm) are shown to improve growth parameters, photosynthetic pigments, IAA contents, yield and antioxidant activity of harvested seeds of fenugreek.^45^

Micronutrient Cu is indispensable for plant growth and tissue culture protocols. Supplementing copper in the form of CuNPs in tissue culture media showed superior results than CuSO_4_ · 5H_2_O in terms of somatic embryogenesis and regeneration.^46^ However, above optimum concentration of CuNPs in the culture media is toxic.^47,48^ Callogenesis and regeneration of seeds explants of rice (*Oryza sativa* L.) were significantly increased by fortification of Chu’s N_6_ media with 10 mg/l CuO-NPs. In contrast to callogenesis, the maximum regeneration of seed explant of rice is achieved at higher concentration (20 mg/l) of CuO-NPs.^48^ Besides, CuNPs and AgNPs are also employed for production of pharmacologically important phenolics, flavonoids, phenylalanine ammonia lyase and antioxidants from callus and suspension culture of plants.^40,49^ Nonetheless, application of nanotechnology in plant tissue culture and biotechnology is controversial and has not been fully recognized, as both positive and negative results are reviewed in literature.^50^

Reports on comparative effects of CuSO_4_, AgNO_3_, CuNPs and AgNPs along with plant growth regulators on tissue culture responses of wheat are rare. Visualizing the massive benefits of metal NPs, present study was therefore conducted to explore potential of AgNPs and CuNPs for callus induction and regeneration efficiency of wheat, and to establish an efficient, reproducible callus induction and regeneration system for mature embryo explants of wheat (*Triticum aestivum* L.). To the best of our knowledge, this is first report highlighting the combined effects of Cu and Ag nanoparticles on tissue culture responses of wheat.

## 2. Results

### 2.1. Characterization of Prepared CuNPs

**Figure 1.**
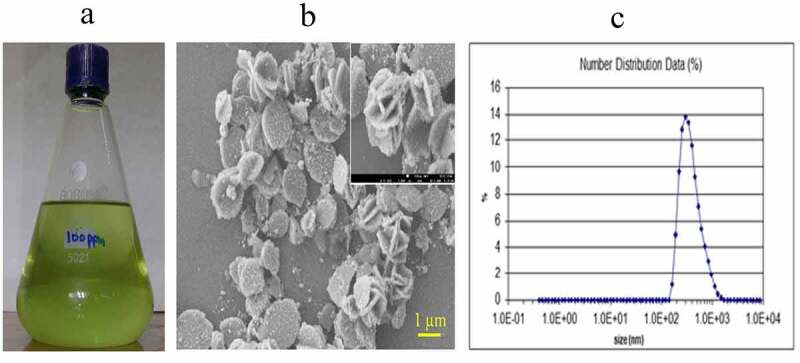
From synthesis to characterization of CuNPs. (a) Stock solution of prepared 100ppm CuNPs. (b) SEM image of CuNPs, (c) Zeta particle analysis of prepared CuNPs.

Zeta potential analysis was used to determine the surface charge of copper nanoparticles in solution. The surface morphology, size and shape of the Copper nanoparticles were analyzed by Zeta analyzer and Scanning Electron Microscope at Nuclear Institute of Biology and Genetic Engineering, (NIBGE) Faisalabad. The synthesized particles through green chemistry were highly homogenous and round in shape having particle size of 20–100 nm (Fig. 1b).

### 2.2. Characterization of Prepared AgNPs

**Figure 2.**
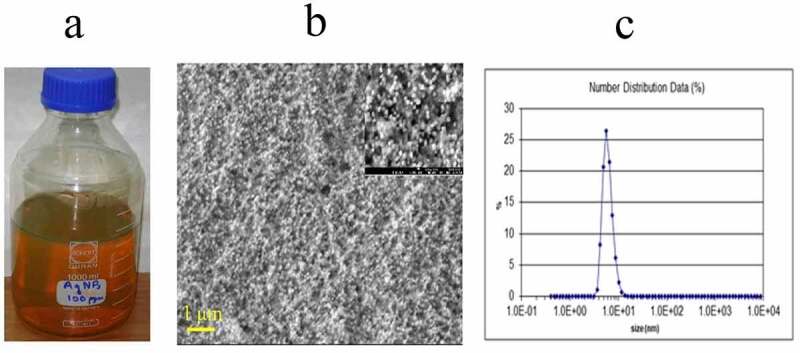
From synthesis to characterization of AgNPs. (a) Stock solution of prepared 100ppm AgNPs. (b) SEM image of AgNPs, (c) Zeta particle analysis of prepared AgNPs.

Zeta potential analysis was used to determine the surface charge of silver nanoparticles in solution. The surface morphology, size and shape of the silver nanoparticles were analyzed by Scanning Electron Microscope at Nuclear Institute of Biology and Genetic Engineering (NIBGE), Faisalabad. The maximum number of AgNPs in solution ranged between 10 and 16 nm (Mean particle size appropriately 12.5 ± 1.5 nm) (Fig. 2a): silver nanoparticles synthesized by reduction of silver nitrate (AgNO3) with tri-sodium citrate (Na_3_C_6_H_5_O_7_.2H_2_O), (Fig. 2b) SEM image of silver nanoparticles and (Fig. 2c) zeta potential analysis of AgNPs]. The SEM images showed individual silver nanoparticles which were predominantly spherical in shape. The SEM image shows the size of the silver nanoparticles ranging from 40 to 50 nm. Almost similar result for size of silver nanoparticles synthesized from Aloe vera extract^51^ and hirta leaves^52^ were reported earlier.

### 2.3. Callus Induction

#### 2.3.1. Optimization of Initial Callus Induction Medium

**Figure 3.**
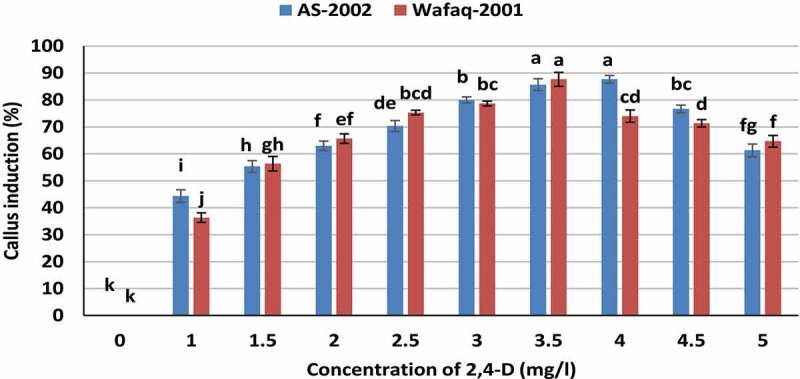
Callogenesis response of wheat genotypes to various induction medium. Bars sharing similar letters do not differ significantly (*p* < .05).

The MS basal media was supplemented with various concentrations of 2,4-D (0, 1.0, 1.5, 2.0, 2.5, 3.0, 3.5, 4.0 and 4.5 mg/l) to standardize the induction media for both genotypes (AS-2002 and Wafaq-2001). Cell division was observed about 72 h after culturing of explants with evident swelling of explants in both genotypes. Our results suggested that presence of auxin (2,4-D) in culture medium is mandatory for primary callus induction from mature embryo explants as no calli were induced in the absence of 2,4-D (Fig. 3). The interaction of concentrations/induction media and genotypes was significant (α = 0.05). Both genotypes AS-2002 and Wafaq-2001 showed maximum callusing potential (85.67% and 87.67%, respectively) in response to MS media supplemented with 3.3 mg/l 2,4-D. However, callus induction of AS-2002 from 3.3 mg/l and 4.0 mg/l 2,4-D did not differ significantly. There was an increase in callus induction frequency with increasing concentration of 2,4-D up to 3.5 mg/l for both genotypes. However, callus induction frequency of genotype Wafaq-2001 significantly declined above 3.5 mg/l 2,4-D (Fig. 3). The mean callus induction potential of genotype AS-2002 and Wafaq-20 in response to various callus induction media was at par with callus induction potential of 62.43% and 61.00%, respectively.

#### 2.3.2. Effect of 2,4-D on Embryogenic Callus Induction

**Figure 4.**
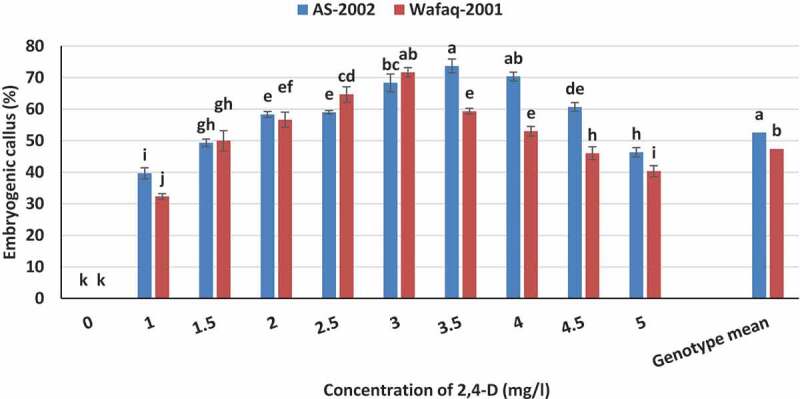
Embryogenic callusing frequency of wheat genotypes in response to various concentrations of 2,4-D. Bars sharing similar letters do not differ significantly (*p* < .05).

We also investigated eﬀects of various concentrations of 2,4-D on embryogenic callus induction during two bi-weekly subcultures of the primary calli. The non-embryogenic calli differentiated into root-type and gradually turned brown and died. After additional one subculture, the embryogenic callus induction frequency was calculated. The results showed that low concentration of 2,4-D (3.5 mg/l) promoted embryogenic callus formation (73.67%) in AS-2002, while 3.0 mg/l was found optimum for Wafaq-2001 with embryogenic callus induction of 71.67% (Fig. 4). The genotypes differed significantly for potential of embryogenic callus induction in response to various concentration of auxin. Genotype AS-2002 yield higher embryogenic calli (mean 52.70%) than Wafaq-2001 (mean 47.40%). Although higher concentrations of 2,4-D could potentially induce a greater number of primary calli (Fig. 3), but it negatively influenced probability of embryogenic embryos (Fig. 4). Therefore, 3.0 and 3.5 mg/l 2,4-D were taken as standard for further optimization studies of Wafaq-2001 and AS-2002, respectively, and to monitor effects of CuSO_4_, AgNO_3_ and their nanoparticles on their tissue culture responses.

#### 2.3.3. Optimization of Regeneration Medium

**Figure 5.**
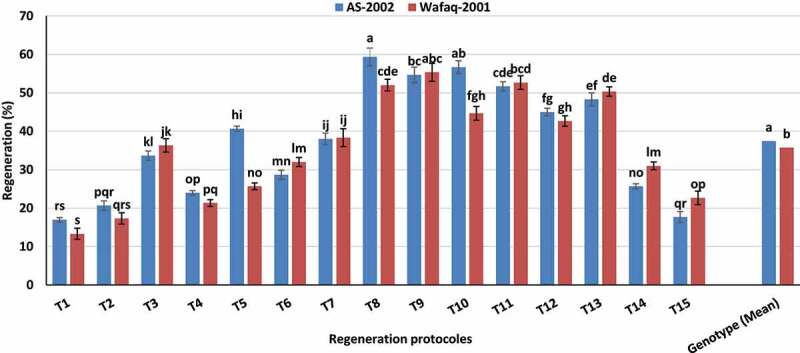
Regeneration response of wheat genotypes to various regeneration protocols. Bars sharing similar letters do not differ significantly (*p* < .05).

The embryogenic calli were induced from mature embryo explants using optimized concentration of 2,4-D (MS-based medium supplemented with 3.0 and 3.5 mg/l 2,4-D for Wafaq-2001 and AS-2002, respectively) and transferred to maintenance medium for proliferation. Some of the calli turned green in maintenance medium. These maintained calli were then transferred to MS-based regeneration medium. We tested fifteen different MS-based regeneration protocols comprising various combinations of BAP, IAA and Kin (Table 1) to establish efficient regeneration system for wheat genotypes (AS-2002 and Wafaq-2001). The regeneration was dependent on interaction of genotypes and regeneration protocols (Fig. 5) Genotype AS-2002 showed maximum regeneration (59.33%) in response to regeneration protocol comprising 0.5 mg/l IAA, 0.3 mg/l BAP and 1.0 mg/l Kin (T_8_; Fig. 5). While, Wafaq-2001 performed best in response to T_9_ (0.5 mg/l IAA, 0.3 mg/l BAP and 1.5 mg/l Kin) with 55.33% regeneration efficiency (Fig. 5). These protocols were taken as standard for further studies comprising CuSO_4_, AgNO_3_ and their nanoparticles. Protocols (T_1_, T_2_, T_4_) comprising higher concentration of auxin (high auxin to cytokinin ratio) and vice versa (T_14_, T_15_) demonstrated poor regeneration frequency in both genotypes. However, at ideal auxin and cytokinins ratio, mainly reduced auxin-to-cytokine ratio (T_8_, T_9_, T_10_ and T_11_) exhibited splendid results. The least regeneration (13.33%) was recorded for genotype Wafaq-2001 on regeneration medium supplemented with 0.5 mg/l IAA and 0.3 mg/l BAP (T_1_) i.e. high auxin-to-cytokinins ratio (Table 1; Fig. 5). Both genotypes differ significantly for regeneration potential (mean values) and the genotype AS-2002 offered better regeneration efficiency (37.44%) than Wafaq-2001 (35.71%).

**Table 1.** Regeneration protocols comprising various combination of IAA, BAP and Kin

Regeneration medium	IAA (mg/l)	BAP (mg/l)	Kin (mg/l)
T_1_	0.5	0.3	0
T_2_	0.5	0.6	0
T_3_	0.5	0.9	0
T_4_	0.5	0	0.5
T_5_	0.5	0	1
T_6_	0.5	0	1.5
T_7_	0.5	0.3	0.5
T_8_	0.5	0.3	1
T_9_	0.5	0.3	1.5
T_10_	0.5	0.6	0.5
T_11_	0.5	0.6	1
T_12_	0.5	0.6	1.5
T_13_	0.5	0.9	0.5
T_14_	0.5	0.9	1
T_15_	0.5	0.9	1.5

### 2.4. Effect of CuSO_4_ and CuNPs on Tissue Culture Responses of Wheat Genotypes

After standardizing primary embryogenic callus induction medium and the regeneration medium for both genotypes, we further investigated effect of CuSO_4_ and CuNPs on tissue culture responses of wheat genotypes. The primary embryogenic callus induction medium (MS-based medium supplemented with 3.0 and 3.5 mg/l 2,4-D for Wafaq-2001 and AS-2002, respectively) and regeneration medium (MS-based regeneration medium comprising 0.5 mg/l IAA, 0.3 mg/l BAP, 1.0 mg/l Kin for AS-2002; and 0.5 mg/l IAA, 0.3 mg/l BAP, 1.5 mg/l Kin for Wafaq-2001) were supplemented with various concentrations of CuSO_4_ and CuNPs (Table 2a) to test and compare their effects on tissue culture responses of both genotypes. The standard MS medium contains 0.025 mg/l CuSO_4_.^16^ Therefore, it was supplemented with optimized concentration of auxin (2,4-D) and cytokine (IAA, BAP and Kin) and was taken as control (Table 2a; C_4_) for comparison.

**Table 2.** Effect of CuSO_4_ and CuNPs on tissue culture responses of wheat genotypes

A			B			C			D		
Treatments			Callus induction (%)			Embryogenic callus (%)			Regeneration (%)		
	CuSO_4_ (mg/L)	CuNPs (mg/L)	AS-2002	Wafaq-2001	**Mean**	AS-2002	Wafaq-2001	**Mean**	AS-2002	Wafaq-2001	**Mean**
C_1_	0.010	-	79.67	82.67	**81.17 g**	68.33	62.67	**65.50 ef**	49.67	50.33	**50.00 ef**
C_2_	0.015	-	81.67	82.67	**82.17 fg**	69.67	70.00	**69.83 cd**	53.33	51.33	**52.33 de**
C_3_	0.020	-	84.67	82.67	**83.67 defg**	74.00	74.00	**74.00 ab**	56.00	54.33	**55.17 cd**
C_4_	MS based medium with standard CuSO_4_ 0.025 mg/l (Control)		86.00	90.33	**88.17 bc**	78.33	73.00	**75.67 a**	59.33	57.00	**58.17 bc**
C_5_	0.030	-	92.00	94.33	**93.17 a**	73.33	68.67	**71.00 bcd**	49.00	44.33	**46.67 f**
C_6_	0.035	-	82.33	91.33	**86.83 bcde**	67.33	64.67	**66.00 ef**	38.67	38.33	**38.50 g**
C_7_	-	0.010	82.33	84.33	**83.33 efg**	69.67	67.67	**68.67 de**	60.00	51.67	**55.83 cd**
C_8_	-	0.015	86.67	84.67	**85.67 cdef**	79.00	74.67	**76.83 a**	65.67	63.00	**64.33 a**
C_9_	-	0.020	87.67	87.67	**87.67 bcd**	73.00	71.33	**72.17 bc**	62.67	58.33	**60.50 ab**
C_10_	-	0.025	89.33	91.67	**90.50 ab**	69.33	67.00	**68.17 de**	51.33	44.00	**47.67 f**
C_11_	-	0.030	88.33	92.67	**90.50 ab**	64.00	62.33	**63.17 f**	37.33	40.33	**38.83 g**
C_12_	-	0.035	82.67	85.67	**84.17 cdefg**	58.00	53.67	**55.83 g**	32.33	30.67	**31.50 h**
Mean			**85.64**	**87.19**		**70.33 a**	**67.47 b**		**51.50 a**	**48.47 b**	
			**LSD value** Genotype ^NS^ = 1.6568 Treatments* = 4.0583 Genotype × Treatments ^NS^ = 5.7393 (*Significant;^NS^ Non-significant) Values sharing common letters do not differ significantly (α = 0.05)			**LSD value** Genotype * = 1.4184 Treatments* = 3.4745 Genotype × Treatments ^NS^ = 4.9136 (*Significant;^NS^ Non-significant) Values sharing common letters do not differ significantly (α = 0.05)			**LSD value** Genotype * = 1.6587 Treatments* = 4.0629 Genotype × Treatments ^NS^ = 5.7459 (*Significant;NS Non-significant) Values sharing common letters do not differ significantly (α = 0.05)		

**Figure 6.**
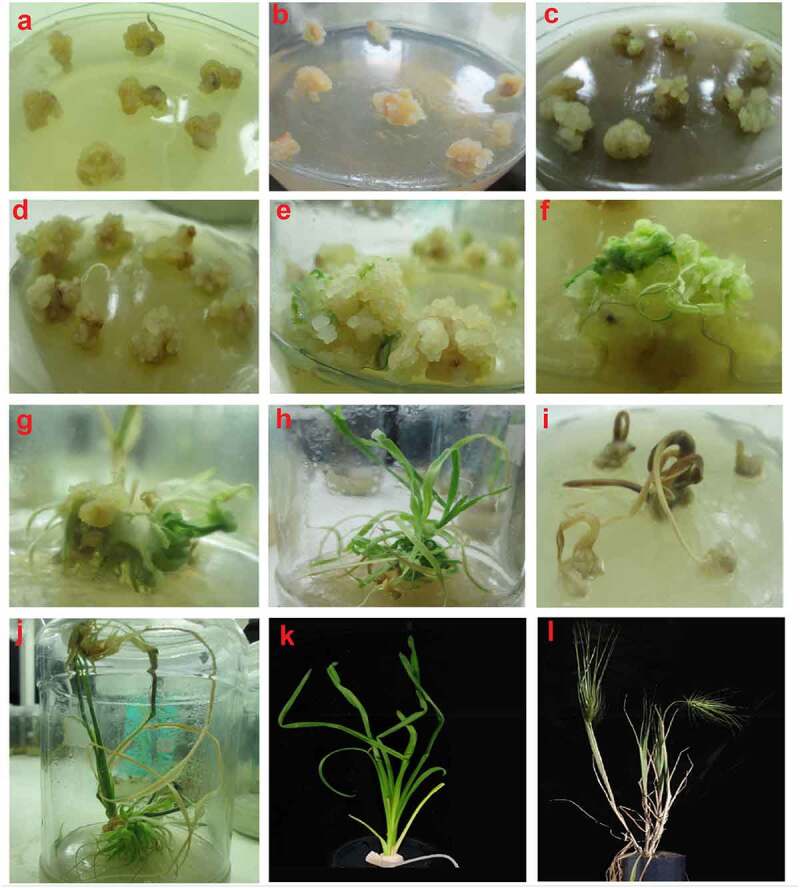
Callus induction and plant regeneration from two wheat cultivars (AS-2002 and Wafaq-2001) and its response to various concentrations of Cu and Ag in salt and nanoparticles form supplemented with growth regulators and hormones. (a) Initial Callus formation (b) callus browning with higher doses of 2,4-D (c) Higher concentration of AgNPs causes callus browning (d) Excess amount of CuNPs promoting callus browning (e) embryogenic callus formation with optimized medium showing green spots (f) Morphology of developing roots (g) Regeneration in response to combined application of 0.015 mg/l CuNPs and 4 mg/l AgNPs (h) Fully developed shoots and branches (i) Toxic effects of Cu and AgNPs causes browning of callus and death of shoot initiation. (j) Fully developed mature plant with multiple shoots and roots system. (k) Regenerated well developed plants in ½ MS medium to improve root system. (l) Normal fertile plants grown in clay pots.

Both genotypes showed almost same response to induction medium fortified with various concentrations of CuSO_4_ and CuNPs. The interactive effect of genotypes and various induction medium on callus induction (%) was non-significant. However, modified induction medium with various concentration of CuSO_4_ and CuNPs showed significant variations for callus induction from mature embryo explants of wheat (Table 2b). Induction medium fortified with C_5_(0.03 mg/l CuSO_4_), C_10_(0.025 mg/l CuNPs) and C_11_ (0.030 mg/l CuNPs) showed non-significant variability and yielded maximum calli with corresponding callusing potential of 93.17%, 90.0% and 90.0%, respectively, then standard concentration of CuSO_4_ i.e. 0.025 mg/l CuSO_4_. It showed that fortification of induction medium with Cu in the form of ions or NPs had analogous effects on callus induction. The sub-optimal concentration of CuSO_4_ (C_1_, C_2_, C_3_) and higher level of CuNPs (C_12_) did yield lower callus frequency. The least callusing frequency was witnessed from MS-based induction medium fortified with C_1_(0.010 mg/CuSO_4_), C_2_(0.015 mg/CuSO_4_), C_3_(0.020 mg/CuSO_4_) and C_12_(0.035 mg/CuNPs) with non-significant differences. CuNPs at higher concentration (0.035 mg/l) accelerated browning of callus and also stimulated appearance of root-like structures, which was detrimental for callus proliferation (Fig. 6d).

The medium fortified with various concentrations of CuSO_4_ and CuNPs confirmed significant effect on embryogenic callus induction, and the genotypes responded differently to CuSO_4_ and CuNPs. Contrary to callus induction, the relatively lower concentration of CuNPs (C_8_) and CuSO_4_ (C_3_ and C_4_) promoted frequency of embryogenic callus. The results showed that addition of either 0.020 mg/l or 0.025 mg/l CuSO_4_ or 0.015 mg/l CuNPs showed comparable results for embryogenic callus induction and were statistically at par with respective embryogenic callus induction frequency of 74.00%, 75.67% and 76.83%. Whereas, concentrations of CuSO_4_ above 0.030 mg/l significantly diminished frequency of embryogenic callus of both genotypes (Table 2c). The calli were induced using standardized induction medium for embryogenic calli for each genotype (Fig. 4). Based on preliminary results (Fig. 5), the basal regeneration medium for genotype AS-2002 comprised 0.5 mg/l IAA, 0.3 mg/l BAP and 1.0 mg/l Kin, while for Wafaq-2001 the media was supplemented with 0.5 mg/l IAA, 0.3 mg/l BAP and 1.5 mg/l Kin except various concentration of copper either in the form of CuSO_4_ or CuNPs (Table 2a). Both genotypes differ significantly for regeneration potential in response to various MS-based regeneration protocols comprising various concentrations of CuSO_4_ and CuNPs. Interestingly, regeneration was higher from medium fortified with CuNPs than CuSO_4_ (Table 2d). Significantly higher regeneration was observed from C_8_(64.33%) and C_9_(60.50%) than C_4_(58.17%), indicating that regeneration of wheat genotypes is significantly increased by substituting CuSO_4_ (standard 0.025 mg/l) with 0.015 mg/l or 0.020 mg/l CuNPs. The results showed that within various treatments of CuSO_4_ (C_1_–C_6_), MS-based regeneration medium fortified with higher or lower concentration of CuSO_4_ than 0.025 mg/l (MS standard) resulted in lower regeneration frequency. Similarly, concentrations of CuNPs above 0.020 mg/l also imparted negative effect on regeneration of wheat mature embryo explants (Table 2d).

### 2.5. Effect of AgNO_3_ and AgNPs on Tissue Culture Response of Wheat

The standardized MS basal induction (MS-based medium supplemented with 3.0 and 3.5 mg/l 2,4-D for Wafaq-2001 and AS-2002, respectively) and regeneration medium (MS-based regeneration medium comprising 0.5 mg/l IAA, 0.3 mg/l BAP, 1.0 mg/l Kin for AS-2002; and 0.5 mg/l IAA, 0.3 mg/l BAP, 1.5 mg/l Kin for Wafaq-2001) for both genotypes comprising standard concentration of CuSO_4_ (0.025 mg/l) was supplemented with various concentration of AgNO_3_ and AgNPs. The induction medium and regeneration medium comprising only standard CuSO_4_ was taken as control (Table 3a).

**Table 3.** Effect of AgNO_3_ and AgNPs on tissue culture responses of wheat genotypes

A				B			C			D		
Treatments				Callus induction (%)			Embryogenic callus (%)			Regeneration (%)		
CuSO_4_ (Standard) mg/L		AgNO_3_ (mg/L)	AgNPs (mg/L)	AS-2002	Wafaq-2001	**Mean**	AS-2002	Wafaq-2001	**Mean**	AS-2002	Wafaq-2001	**Mean**
S_1_	MS based medium with standard CuSO_4_ 0.025 mg/l (Control)			82.33	84	**83.17 fg**	67	62.33	**64.67 f**	56.67	55.67	**56.17 f**
S_2_	0.025	1	-	81.67	83.67	**82.67 g**	68.33	63.33	**65.83 ef**	57.67	57.67	**57.67ef**
S_3_	0.025	2	-	82.67	86.00	**84.33 fg**	67.00	67.00	**67.00 ef**	60.00	60.00	**60.00cde**
S_4_	0.025	3	-	83.67	88.00	**85.83 defg**	69.67	69.33	**69.50 de**	62.00	61.33	**61.67bcd**
S_5_	0.025	4	-	84.67	88.67	**86.67 cdef**	73.33	75.33	**73.33 cd**	64.33	61.00	**62.67bc**
S_6_	0.025	5	-	89.33	91.33	**90.33 abc**	75.00	71.67	**74.33 bc**	65.33	64.00	**64.67ab**
S_7_	0.025	6	-	90.333	95.67	**93.00 a**	73.00	72.67	**72.83 cd**	59.33	56.67	**58.00ef**
S_8_	0.025	7	-	87.33	94.67	**91.00 ab**	66.67	67.00	**66.83 ef**	47.00	42.67	**44.83 g**
S_9_	0.025	-	1	83.33	83.33	**83.33 fg**	68.67	65.33	**67.00 ef**	60.33	58.67	**59.50de**
S_10_	0.025	-	2	83.33	84.33	**83.83 fg**	71.00	68.33	**69.67 de**	63.00	60.67	**61.83bcd**
S_11_	0.025	-	3	84.67	86.67	**85.67 efg**	77.67	77.33	**77.50 ab**	68.33	65.00	**66.67a**
S_12_	0.025	-	4	86.00	92.00	**89.00 bcde**	80.67	79.00	**79.83 a**	61.33	63.00	**62.17bcd**
S_13_	0.025	-	5	92.00	94.33	**93.17 a**	72.00	72.67	**72.33 cd**	56.33	54.33	**55.33 f**
S_14_	0.025	-	6	89.33	90.00	**89.67 abcd**	69.00	64.33	**66.67 ef**	47.67	44.67	**46.17 g**
S_15_	0.025	-	7	85.67	87.00	**86.33 defg**	62.33	56.33	**59.33 g**	41.00	37.33	**39.17 h**
Mean				**85.76 b**	**88.64 a**		**70.73 a**	**68.82 b**		**58.02 a**	**56.18 b**	
				**LSD value**: Genotype* = 1.4554 Treatments* = 3.9858 Genotype × Treatment = 5.6367 (*Significant;^NS^ Non-significant) Values sharing common letters do not differ significantly (α = 0.05)			**LSD value**: Genotype* = 1.4479 Treatments* = 3.9652 Genotype × Treatment ^NS^ = 5.6077 (*Significant;^NS^ Non-significant) Values sharing common letters do not differ significantly (α = 0.05)			**LSD value**: Genotype* = 1.1041 Treatments* = 3.0238 Genotype × Treatment ^NS^ = 4.2763 (*Significant;^NS^ Non-significant) Values sharing common letters do not differ significantly (α = 0.05		

The induction medium fortified with various concentration of AgNO_3_ and AgNPs promised significant effect on callus induction potential of wheat genotypes. Genotype Wafaq-2001 expressed more callusing potential (88.64%) than AS-2002 (85.76%) in response to various concentrations of AgNO_3_ and AgNPs. Induction medium supplemented with S_6_(5 mg/l AgNO_3_), S_7_(6 mg/l AgNO_3_), S_8_(7 mg/l AgNO_3_), S_13_(5 mg/l AgNPs) or S_14_(5 mg/l AgNPs) exhibited non-significant variations and were found to be promising for maximal callus induction frequency ranging from 90.33% to 93.17% (Table 3b). The reduced concentration of AgNO_3_ or AgNPs (1–3 mg/l) were found ineffectual to improve callus induction potential of wheat genotypes compared with control (basal induction medium deprived of AgNO_3_ and AgNPs; Table 3b). Contrasting to callus induction, when the calli were proliferated in the same refreshed induction medium, maximum embryogenic calli were obtained from the medium fortified with 3 mg/l or 4 mg/l AgNPs (S_11_ and S_12_, respectively) compared with control and rest of the treatments of AgNO_3_ (Table 3c). Higher concentration of AgNPs (7 mg/l) significantly declined the frequency of embryogenic callus (59.33%) compared with control (64.67%). The higher concentrations of AgNO_3_ and AgNPs (6–7 mg/l) accelerated browning of embryogenic callus, stimulated appearance of root-like structures in both genotypes and were maleficent to embryogenic callus proliferation (Table 3c).

The wheat genotypes differed significantly for regeneration potential in response MS-based regeneration medium fortified with AgNO_3_ and AgNPs. Genotype AS-2002 displayed higher regeneration efficiency (58.02%) than Wafaq-2001 (56.18%). The regeneration medium fortified with 5.0 mg/l AgNO_3_ or 3 mg/l AgNPs (S_6_ and S_11_, respectively) showed pronounced effect on regeneration of wheat genotype and yielded maximum regenerants compared with control (S_1_) i.e. media devoid of silver in the form of AgNO_3_ or AgNPs (Table 3d). Non-significant difference was observed for regeneration within both treatments (S_6_ and S_11_); however, 3 mg/l AgNPs yield more regenerants (66.67%) than 5 mg/AgNO_3_ (64.67%). Akin to effect of higher concentration of AgNO_3_ and AgNPs on embryogenic callus induction, their higher levels (S_8_, S_14_ and S_15_) also imparted negative effect on regeneration and yielded lower regeneration frequency than control (Table 3d).

### 2.6. Effect of Combined Application of CuNPs and AgNPs on Tissue Culture Responses of Wheat

We also tested individual and combined effect of CuNPs and AgNPs on tissue culture responses of wheat. From preceding experiments, treatments of CuNPs and AgNPs which yielded maximum embryogenic calli and confirmed increased regeneration were selected. Therefore, treatments C_7_(0.010 mg/l), C_8_(0.015 mg/l) and C_9_(0.020 mg/l) for CuNPs (Table 2) and S_10_(2 mg/l), S_11_(3 mg/l) and S_12_(4 mg/l) for AgNPs (Table 3) were selected for this part of the study. The MS-based induction medium was fortified with appropriate concentration of 2,4-D (3.0 mg/l and 3.5 mg/l for Wafaq-2001 and AS-2002, respectively) for callus induction of each genotype (Fig. 4). While, regeneration medium was supplemented with appropriate concentration of auxin and cytokinins (0.5 mg/l IAA, 0.3 mg/l BAP, 1.0 mg/l Kin for AS-2002; and 0.5 mg/l IAA, 0.3 mg/l BAP, 1.5 mg/l Kin for Wafaq-2001). We did not use CuSO_4_ for protocols comprising CuNPs. The MS-based regeneration medium comprising standard 0.025 mg/l CuSO_4_^16^ with standardized concentration of auxin and cytokinins was taken as control (Table 4a).

**Table 4.** Effect of various combinations of CuNPs and AgNPs on tissue culture responses of wheat genotypes

A				B			C			D		
Treatments				Callus induction (%)			Embryogenic callus (%)			Regeneration (%)		
	CuSO4 (mg/l)	CuNPs (mg/l)	AgNPs (mg/l)	AS-2002	Wafaq-2001	**Mean**	AS-2002	Wafaq-2001	**Mean**	AS-2002	Wafaq-2001	**Mean**
N_1_	0.025 mg/l (Control)			85.00	85.00	**85.00 defg**	74.67	71.67	**73.17 defg**	59.33	56.33	**57.83 efgh**
N_2_		0.010		81.33	82.67	**82.00 g**	71.33	67.67	**69.50 g**	54.33	57.67	**56.00 gh**
N_3_		0.015		86.33	88.67	**87.50 bcd**	77.33	73.67	**75.50 bcd**	62.68	60.67	**61.67 cd**
N_4_		0.020		83.00	83.33	**83.17 efg**	72.00	72.33	**72.17 defg**	58.67	57.67	**58.17 defgh**
N_5_	0.025		2	85.67	88.67	**87.17 cde**	75.67	74.00	**74.83 cd**	62.00	56.67	**59.33 cdefg**
N_6_	0.025		3	84.00	91.33	**87.67 bcd**	77.33	78.67	**78.00 abc**	63.67	62.00	**62.83 bc**
N_7_	0.025		4	90.00	92.67	**91.33 ab**	81.68	79.33	**80.50 a**	67.33	65.00	**66.17 b**
N_8_		0.010	2	82.33	85.33	**83.83 defg**	69.33	69.00	**69.17 g**	55.67	53.33	**54.50 h**
N_9_		0.010	3	80.00	86.33	**83.17 efg**	74.00	67.00	**70.50 efg**	58.67	53.67	**56.17 gh**
N_10_		0.010	4	81.00	84.67	**82.83 fg**	71.67	68.33	**70.00 fg**	60.00	56.67	**58.33 defg**
N_11_		0.015	2	90.00	91.00	**90.50 abc**	80.33	71.33	**75.83 bcd**	62.33	59.67	**61.000 cde**
N_12_		0.015	3	84.33	90.00	**87.17 cde**	78.00	71.67	**74.00 cdef**	65.67	66.67	**66.17 b**
N_13_		0.015	4	90.00	95.00	**92.50 a**	84.67	78.00	**80.33 a**	71.67	68.33	**70.00 a**
N_14_		0.020	2	93.67	87.33	**90.50 abc**	77.33	81.00	**79.17 ab**	62.67	61.67	**62.17 c**
N_15_		0.020	3	86.67	87.00	**86.83 cdef**	77.67	74.33	**76.00 bcd**	62.33	60.33	**61.33 cde**
N_16_		0.020	4	83.33	88.00	**85.67 defg**	76.00	72.67	**74.33 cde**	58.67	56.00	**57.33 fgh**
				**85.42 b**	**87.94 a**		**75.96 a**	**73.17 b**		**61.81 a**	**59.31 b**	
				**LSD value**: Genotype* = 1.4327 Treatments* = 4.0523 Genotype × Treatment ^NS^ = 5.7308 (*Significant;^NS^ Non-significant) Values sharing common letters do not differ significantly (α = 0.05)			**LSD value**: Genotype* = 1.4614 Treatments* = 4.1335 Genotype × Treatment ^NS^ = 5.8457 (*Significant;^NS^ Non-significant) Values sharing common letters do not differ significantly (α = 0.05)			**LSD value**: Genotype* = 1.3448 Treatments* = 3.8036 Genotype × Treatment ^NS^ = 5.3791 (*Significant;^NS^ Non-significant) Values sharing common letters do not differ significantly (α = 0.05)		

The callus induction potential of wheat genotypes (AS-2002 vs. Wafaq-2001) differed significantly in response to individual and combined application of Cu and Ag nanoparticles (Table 4a). Genotype Wafaq-2001 offered significant higher callus induction (87.94%) than AS-2002 (85.42%). Comparison of standard CuSO_4_ (control) with sole application of CuNPs (N_2_, N_3_ and N_4_) showed that individual use of CuSO_4_ (0.025 mg/l) or CuNPs (0.010–0.020 mg/l) did not differ significantly for callus induction. However, callusing was increased when AgNPs were supplemented in the MS-based induction medium along with standard CuSO_4_ (0.025 mg/l) compared with control (only CuSO_4_ @ 0.025 mg/l). Combined application of Cu and Ag nanoparticles in the induction medium also improved callusing frequency and the maximum callus induction was achieved by their co-application i.e. N_11_, N_13_ or N_14_ with callus induction frequency of 90.50%, 92.50% and 90.50%, respectively. However, co-application of higher doses of CuNPS and AgNPs (N_15_ and N_16_) declined callus induction potential of wheat genotypes (Table 4b).

The wheat genotypes exhibited significant difference for embryogenic callus induction potential in response to various combinations of Cu and Ag nanoparticles (Table 4c). Genotype AS-2002 yielded more embryogenic calli (75.96%) than Wafaq-2001 (73.17%). Compared with CuSO_4_, the eﬀects of CuNPs were mainly observed on the number of embryogenic calli producing green shoots, although the difference was not signiﬁcant. The greener spots were observed on calli incubated on N_3_. It was noted that among sole application of Cu either in the form of CuSO_4_ or CuNP, 0.015 mg/l CuNPs (N_3_) yielded more embryogenic callus than standard CuSO_4_ (N_1_ = 0.025 mg/l). Similarly, individual effect of AgNPs (N_5_ – N_7_) along with 0.025 mg/l CuSO_4_ also provoked embryogenic callus formation from mature embryo of wheat, and the treatment comprising 4 mg/l AgNPs (N_7_) exhibited auspicious results with embryogenic callus induction of 80.50% (Table 4c).

Interestingly, co-application of metallic NPs significantly improved embryogenic callus formation of wheat genotypes. The results indicated that precise combination of both nanoparticles (N_8_–N_16_) significantly improved embryogenic callus formation. Induction medium supplemented with N_13_ (0.015 mg/l CuNPs + 4 mg/l AgNPs) or N_14_(0.020 mg/l CuNPs + 2 mg/l AgNPs) showed best results compared with control and other treatments (Table 4c). The frequency of embryogenic calli achieved form N_7_, N_13_ or N_14_ was statistically at par (α = 0.05). Variability in morphology of callus was also noted in response to different concentrations of nanoparticles. The color of calli induced from induction medium supplemented with low concentration of nanoparticles (N_13_) was creamy yellowish with friable textures. Brown granular calli but with stunted growth were observed from induction medium supplemented with high concentration of nanoparticles N_16_ (Fig. 6i).

Significantly higher regeneration was offered by genotype AS-2002 than Wafaq-2001 in response to various treatments/combinations of Cu and Ag nanoparticles (Table 4d). Comparison of individual effect of copper used either in the form of ion or nanoparticles (N_1_, N_2_, N_3_ and N_4_) showed that CuNPs at 0.015 mg/l outdid CuSO_4_ (control) and rest of the treatments. Individual application of AgNPs also improved regeneration potential of wheat and the maximum regeneration was observed in response to 4 mg/l AgNPs (66.17%). Interestingly, co-application of CuNPs and AgNPs significantly improved regeneration efficiency then rest of the treatments. Among all treatments, C_13_(0.015 mg/l CuNP + 4 mg/l AgNPs) exhibited the highest regeneration efficiency (70%) with an increment of 21% over control. We observed that callus induction, embryogenic callusing and regeneration of mature was genotype dependent, where Wafaq-2001 exhibited higher callus induction while AS-2002 showed higher embryogenic callusing and regeneration potential (Table 4(b-d))

## 3. Discussion

### 3.1. Background

In wheat (*Triticum aestivum* L.), immature embryos explant are extensively used to initiate cultures owing to their fabulous regeneration capacity.^3,5,9,53^ Unfortunately, immature embryo explants are not always desired due to their temporal availability and production requirements. On the contrary, mature embryos are easily stored and are readily available as mature seeds throughout the year for callus-based genetic transformation. Therefore, in present study mature embryo explants were employed to study effects of CuSO_4_, AgNO_3_, and their nano particles, i.e., copper nanoparticles (CuNPs) and silver nanoparticles (AgNPs), respectively, on tissue culture responses of wheat.

Among cereals, wheat is the most recalcitrant to genetic transformation due to poor regeneration potential of transformed cell lines.^5,9^ Contrasting to other monocots, not all wheat genotypes are responsive to tissue culture procedures mainly because of deficit secondary growth through cambium tissues and differences in cytoplasmic composition of the cells. Conditions promising for callogenesis and regeneration of one genotype are not always conducive for another genotype of the same species.^54,55^ Hence, wheat genotypes differ for callus induction and regeneration potential, and almost immature embryo of all genotypes had the potential of callogenesis to some extent.^11,14,55^ We employed two wheat genotypes (AS-2002 and Wafaq-2001) and observed that the genotypes differ for tissue culture responses especially for embryogenic callus induction and regeneration in response to media supplemented with various combination of growth regulators (Figs 4and5 &),CuSO_4_, AgNO_3_ and their nano particles (Tables 2, 3 and 4). It is likely that tissue culture responses of genotypes might vary due to dissimilar cytoplasmic composition and gene action.

### 3.2. Auxins Mediated Callus Induction Optimization

Callus growth and development are inﬂuenced by complex relationship between explants and composition of culture medium. The plant growth regulator “auxins” are obligatory for callus induction. Whereas, they had negative effect on regeneration and are therefore omitted or reduced from regeneration medium. Our data suggested that addition of 2,4-D in culture medium is essential for callus induction and embryogenic callus formation from mature embryos. The optimal level of 2,4-D for genotype AS-2002 was 3.5–4.0 mg/l, and 3.5mg/l for Wafaq-2001 for profuse callus induction from mature embryo explants (Fig. 3). We noted that higher concentration of auxin (2,4-D) promoted appearance of root hair-like structures in calli which gradually turned ﬂavescent. Contrarily, lower concentration of 2,4-D (3.5 mg/l) promoted embryogenic callus formation (Fig. 4). Although higher concentrations of 2,4-D could potentially induce a greater number of primary calli, the high doses of 2,4-D could have also resulted in an increased probability of somatic mutation.^56^ Almost, similar results were observed by Mendozza and Kaeppler^15^ in wheat mature embryo culture. Our results are also in accordance with those reported by Mahmood and Razzaq,^57^ and Yadav et al.^9^ Based on results, induction medium supplemented with 3.0 mg/l 2,4-D for Wafaq-2001 and 3.5 mg/l 2,4-D for AS-2002 were found ideal, and taken as standard for further optimization studies (Fig. 4).

We found that concentration of auxins is genotypic dependent and prolific callusing happens only when precise concentrations of auxins are supplemented in the induction media for each genotype (Fig. 3). Normally, wheat genotypes differ for callus induction potential and callusing did happen in all wheat genotypes on induction media supplemented with 2–6 mg/l 2,4-D.^9,11,13^ However, profuse callus induction is dependent on interaction of concentration of growth regulators and genotypes. Earlier, it was found that genotype AS-2002 and GA-2002 produced maximum calli from immature embryo on medium comprising 4 mg/l of 2,4-D; while genotype chakwal-50 yielded maximum number of calli in response to 6 mg/l 2,4-D.^13^ Our results are in agreement with those of Jasdeep et al.,^11^ who reported that wheat genotypes differ for callus induction potential and callusing frequency of genotypes is dependent on concentration of growth regulators.

### 3.3. Regeneration Optimization – Auxins and Cytokinins

Auxins and cytokinins are most important growth modulator and their interaction is crucial for regeneration of embryogenic callus. Different genotypes of wheat behaved differently at various concentrations of phytohormones. Regeneration of cereals is dependent on concentrations of auxins (IAA, 2,4-D and picloram) and cytokinins (kinetin, BAP, zeatin) and their interactions.^9,11,13,58^ The morphogenic calli can be enforced to regenerate shoots by increasing concentration of cytokinins or decreasing auxins in culture media.^13^ Our results showed that both genotypes (AS-2002 and Wafaq-2001) differ for embryogenic callus and regeneration potential. The frequency of embryogenic calli and regeneration of both genotypes were dependent on composition of culture media (Figs. 4 & Figs. 5). The differential regeneration potential of genotypes may be associated with variability in level of endogenous growth hormones and variable gene action controlling organogenesis. Previous studies suggested that wheat genotypes differ in regeneration potential and respond differently to various concentrations of auxins and cytokinins^9,11,57^ reported that MS medium supplemented with 0.4 mg/l 2,4-D, 1.0 mg/l zeatin and 1.0 mg/l benzylaminopurine (BAP) significantly improve regeneration potential of mature embryos of Pakistani wheat genotypes.^13^ identified that 0.2 mg/l IAA, 0.5 mg/l Kin and 0.5 mg/l of BAP is suitable to achieve maximum regeneration of immature embryo of wheat. Similarly, MS media supplemented with 2.0 mg/l kinetin, 0.5 mg/l NAA and 0.5 mg/l BAP is accepted for maximum shoot regeneration of Indian wheat genotypes.^11^

### 3.4. Effect of CuSO_4_ and AgNO_3_ on Tissue Culture Response of Wheat

The CuSO_4_ (0.025 mg/l) is an integral component of MS medium.^16^ In plants, copper (Cu) acts as essential cofactor of numerous proteins. As a micronutrient, a minimal amount of Cu is necessary for plants. However, excess of Cu may exert in contrast detrimental effects on cellular functioning and plant survival.^17^ We found that MS medium fortified with various concentrations of CuSO_4_ confirmed significant effect on tissue culture responses of mature embryo explants of wheat. CuSO_4_ at 0.025 mg/l was found optimum for efficient embryogenic callus and regeneration against reduced (0.010–0.015 mg/l) and higher concentration (0.035 mg/l) of CuSO_4_ (Table 2). CuSO_4_ (0.1–100 μM) had the potential to significantly increase shoot regeneration from callus of leaf disc of wheat, triticale and tobacco.^18^ In sorghum, regeneration potential of the explants was significantly reduced on MS media fortified with kinetin and IAA but devoid of CuSO_4_. Conversely, regeneration was dramatically increased when Cu (2 mM) was supplemented along with kinetin (9.2 mM) and IAA (2.85 mM).^59^ Our results are supported by Kumar et al.^14^ who found that incubation of calli on media supplemented with 2,4-D, zeatin and CuSO_4_ significantly enhance multiple shoot induction in wheat. Purnhauser and Gyulai^18^ had also reported that culture media fortified with CuSO_4_ and AgNO_3_ can potentially augment shoot regeneration from calli of triticale and wheat.

We also found that 5–7 mg/l AgNO_3_ significantly improves the callus induction potential of mature embryo explants of wheat, whereas 5.0 mg/l AgNO_3_ yielded maximum embryogenic callus and regenerants (Table 3). Addition of AgNO_3_ (1.0 mg/l) in the media does increase somatic embryogenes of immature embryo culture of durum wheat.^60^ During *in vitro* culture of plant tissues, ethylene is produced by plant tissues and may accumulate to toxic level in the culture vessels, particularly from rapidly growing non-differentiated callus or suspension cultures. The ethylene action can be inhibited by supplementing silver ions (Ag^+^) in the culture media. It is well established that AgNO_3_ is very potent inhibitor of ethylene action and is widely used in callus induction and regeneration media ^(9,25,26,50)^ to counteract the ethylene precursors like 1-aminocyclopropane-1-carboxylic acid (ACC) and 2-chloroethylphosphonic acid.^42^ However, the extent of the response and the optimum concentration of AgNO_3_ are cultivar dependent.^61^

AgNO_3_ is normally employed along with auxins and cytokinins to improve callus induction and regeneration of plants. For instance, callus induction frequency of tomato was improved by addition of AgNO_3_ in MS basal media along with IAA and BAP. The regeneration potential of potato was increased with co-application of 0.1 mg/l IAA, 1.0 mg/l zeatin, 2.0 mg/l BAP and 8–10 mg/l AgNO_3_.^25^ Also, supplementing culture media with silver ions in the form of AgNO_3_ had provoked callus induction from immature embryo explants of maize.^20^ Similarly, in rice somatic embryogenesis and regeneration of mature embryo explants were increased in response to addition of AgNO_3_ in MS-based induction and regeneration media.^21^ A number of dicotyledonous species including, pomegranate,^62^ cucumber^23^ and pearl millet^63^ had also offered significant improvement in regeneration with addition of AgNO_3_ in the media and thus support our results.

### 3.5. Employment of Nanoparticles to Improve Callus Induction and Regeneration

The research on possible role of nanoparticles of Cu and Ag in plant tissue culture is limited. Preliminary reports suggested that Cu and Ag nanoparticles indirectly increase tissue culture responses of explants owing to wide range of antimicrobial activity including fungi, gram-negative and gram-positive bacteria.^64,65^ Therefore, establishment of tissue culture protocols was mainly restricted to standardization of plant growth regulators in the tissue culture media. We found that addition of CuNPs at 0.025–0.030 mg/l in the media significantly improved callusing frequency, whereas maximum embryogenic callus induction and regeneration were achieved in response to 0.015 mg/l CuNPs and 0.015–0.020 mg/l CuNPs, respectively (Table 2). Due to monoclinic structure, CuNPs had antimicrobial and excellent antioxidant properties.^66^ The positive eﬀects of CuNPs on callus induction might be because copper is an essential nutrient for plants growth, acts as a cofactor in many metallo-proteins and is structural element of regulatory proteins.^17^ Copper is also involved in important physiological processes like electron transport chain, hormone signaling and cell wall metabolism.^67^ At higher doses of CuNPs (C_12_) decrease in callogenesis, embryogenic calli and regeneration were witnessed (Table 2). It might be associated with facts that high concentrations of NPs prove toxic for living system, since release of Cu ions from CuNPs induces oxidative stress by catalyzing formation of (OH^−^) radicals from non-enzymatic chemical reaction between superoxide and H_2_O_2_.^68^ Chang et al.^69^ had reported that CuNPs can be toxic at higher concentrations, so use of CuNPs in tissue culture should not exceed physiological tolerance range. Anwaar et al.^48^ observed that seeds explants of rice significantly responded to the addition of CuONPs in Chu’s N_6_ medium comprising 1.0 mg/l NAA, 0.5 mg/l BAP, and 0.5 mg/l kinetin. They reported that callus induction frequency increases only up to concentration of 10 mg/l CuONPs while regeneration up to 20 mg/l of CuONPs. Optimum concentration of CuNPs had also positive effects on clonal micro reproduction of *Mentha longifolia*.^70^

The CuNPs at 0.015 mg/l (C_8_) showed better results than their salt (0.025 mg/l CuSO_4_) for regeneration (Table 2). It indicated that regeneration of mature embryo explants of wheat can be improved by substituting 0.025 mg/l CuSO_4_ in the MS medium with 0.015 mg CuNPs. The comparative effect of CuNPs and CuSO_4_ on somatic embryogenesis and regeneration of *Ocimum basilicum* had validated our results.^46^ Where it is reported that inclusion of CuNPs (5 µM) significantly improves somatic embryogenesis and regeneration in comparison to the control treatment (0.1 µM CuSO_4_ · 5H_2_O), confirming that use of copper as CuNPs is superior to CuSO_4_ · 5H_2_O.^46^ Also, addition of CuNPs in induction medium had also showed positive effect on growth indices of regenerated plants like plant height, growth index, quantity of internodes, quantity of shoots and reproduction coefficient of *Mentha longifolia*.^70^

AgNPs are chemically more reactive than silver ions because of their higher surface area-to-mass ratio.^50^ As an alternative to AgNO_3_ we tested silver nanoparticles (AgNPs) to access their role in tissue culture responses of wheat. We noted that 5.0 mg/l AgNPs significantly improve callus induction potential of mature embryo explants of wheat (Table 3). Similarly, 3–4 mg/l AgNPs induced maximum embryogenic calli compared with control and rest of the treatments (Table 3). Relatively lower concentration of AgNPs (3 mg/l) was found promising than higher concentration of AgNPs (4–7 mg/l) for regeneration. We found that AgNPs at 3 mg/l offered maximum and statistically equal regeneration frequency to that of 5 mg/l AgNO_3_ (Table 3). It reveals that fortification of tissue culture media with silver nanoparticles (AgNPs) improves vigor and regeneration frequency of explants, mainly because of higher ability of silver ions to inhibit ethylene biosynthesis.^43,50,71^ Manickavasagam et al.^72^ reported that biosynthesized silver nanoparticles when supplemented in tissue culture media promote callus induction frequency, callus regeneration and rhizogenesis along with significant decrease in concentration of reactive oxygen species, hydrogen peroxide and malondialdehyde in *Menthalongi folia*. A significant increase in callus induction and callus growth rate of *Phaseolus vulgaris* is reported in response to exposure of explants to 50 mg/l AgNPs for 30 min, while higher concentration were found detrimental for callogenesis.^73^ Fresh weight of callus is improved by silver nanoparticle treatments probably due to increased activities of antioxidant enzymes and reduced levels of reactive oxygen species.^74^

### 3.6. Combined Effects of CuNPs and AgNPs on Tissue Culture Response of Plants

Our results suggested that co-application of CuSO_4_ or CuNPs with AgNPs improve frequency of embryogenic callus and regeneration of mature embryo explants of wheat (Table 4). Co-application of 0.015 mg/l CuNPs with 4 mg/l AgNPs (N_13_) or 0.020 CuNPs with 2 mg/l AgNPs (N_14_) in the induction media yielded maximum embryogenic calli (Table 4). Similarly, co-application of CuNPs (0.015 mg/l) with AgNPs (4 mg/l) along with optimized concentration of auxin and cytokinins improved regeneration of mature embryo explants up to 21% over control (Table 4). The results showed that Cu and Ag nanoparticles had definite role in dedifferentiation and regeneration of mature embryo explant of wheat. The increased regeneration of morphogenic calli in response to co-application of appropriate concentration of NPs might be associated with their interactive effects to reduce ethylene action, excellent antioxidant activity and better bio-acceptance^25,42,43^ in favor of important physio-biochemical processes associated with regeneration.^75^ We found that treatments comprising higher concentrations of both NPs imparted negative effects on callogenesis and regeneration of mature embryo explant of wheat (Table 4), showing that higher concentrations of NPs are always toxic for living system. Since, excessive concentration of CuO-NP releases Cu ions which induces oxidative stress on living tissues.^68^

## 4. Materials and Methods

### 4.1. Synthesis and Characterization of Nano Particles

#### 4.1.1. Synthesis of Silver Nanoparticles (AgNPs)

Silver nanoparticles (AgNPs) were synthesized by reduction of silver nitrate (AgNO_3_) with tri-sodium citrate (Na_3_C_6_H_5_O_7_.2H_2_O) according to the method reported by^76^ with slight modifications as below:

3AgNO_3_ + Na_3_C_6_H_5_O_7_.2H_2_O → C_6_H_5_O_7_ + 3NaNO_3_ + CO_2_ + 3Ag+2H_2_O

Silver nitrate (510 mg) was dissolved in 500 ml distilled water and heated for 15 minutes at 75–80°C with continuous stirring at 7000 rpm on magnetic stirrer. Then 500 ml solution containing 300 mg of tri-sodium citrate was added slowly. The solution was kept at 75–80°C with continuous stirring for about an hour. When the solution turned golden yellow the reaction was stopped (indication of silver nanoparticles). To it 1.0 mg/l ascorbic acid was added as capping agent to stabilize the nanoparticles. Furthermore, reaction conditions and concentration of the reactants were adjusted in such a way to ensure that no silver ions were left in the solution. Molar mass of AgNO_3_ and Na_3_C_6_H_5_O_7_ depending upon purity was so adjusted to finally prepare stock solution of 100 ppm AgNPs.

#### Characterization of AgNPs

Size of AgNPs was determined by Zeta Particle Analyzer and scanning electron microscope (SEM) at Nuclear Institute of Biology and Genetic Engineering (NIBGE), Faisalabad. The synthesized silver nanoparticles were centrifuged at 10,000 rpm for 15 min and the pellets were re-dispersed in sterile double distilled water and centrifuged at 10,000 rpm for 10 minutes. The purified pellets were dried at 50°C in an oven and thin films of dried samples were prepared on a carbon coated copper grid by dropping a very small amount of the samples on the grid. Extra solutions of the samples were removed using a blotting paper. The films on the carbon coated copper grid (SEMgrid) were allowed to dry by putting them under a mercury lamp for 5 min. The morphological features including micrograph images, size, and structure of synthesized nanoparticles were analyzed and recorded.

#### 4.1.2. Synthesis of Copper Nanoparticles (CuNPs)

Copper nanoparticles (CuNPs) were synthesized by biological reduction method using organic extract. A solution of CuSO_4_.5H_2_O was prepared in distilled water and reduced stepwise by addition of 250 ml of onion extract with continuous stirring (3000–4000 rpm) by magnetic stirrer at 100°C in water bath. After an hour, the color of the solution turned translucent yellowish green color which was indication of conversion of Cu^+^ into CuNPs. To it, 5.0 mg of ascorbic acid was added to terminate the reaction. Ascorbic acid acts as a capping agent to stabilize the nanoparticles. Furthermore, reaction conditions and concentration of the reactants were adjusted in such a way to ensure that no Cu ions were left in the solution. Molar mass of CuSO_4_.5H_2_O depending upon purity was so adjusted to finally prepare stock solution of 100 ppm CuNPs.

#### Characterization of Biosynthesized CuNPs

Size of Cu-NPs was determined by Zeta Particle Analyzer and scanning electron microscope (SEM) at Nuclear Institute of Biology and Genetic Engineering (NIBGE), Faisalabad. SEM analysis was carried out by gold coating CuNPs.

#### Stability of Nanoparticles

The main factors that affect the use of CuNPs and AgNPs are their stability in the dispersion. Many capping agents such as Polyvinyl Pyrrolidone (PVP) and Polyethylene glycol are used to prevent agglomeration. Ascorbic acid was used as capping agent in this study to stop the further reaction and avoid contamination of other compounds. The prepared Cu NP suspensions were placed without any further mixing or treatment for 12 weeks, no sedimentation was observed during this period. This indicates that the high capping power of Ascorbic acid for nanoparticles.

### 4.2. Tissue Culture Studies

A series of experiments were conducted to study the effects of CuSO_4_, AgNO_3,_ copper nano particles (CuNPs) and silver nano particles (AgNPs) on tissue culture responses of two wheat genotypes (AS-2002 and Wafaq-2002) employing mature embryo explants. In first step of study, the indication and regeneration medium for each genotype were standardized. In the second step, the standardized basal induction and regeneration media for each genotype were supplemented with CuSO_4_ and CuNPs to study their effect on tissue culture responses of both genotypes (Table 2–A). In the third step, the standardized basal induction and regeneration media for each genotype were supplemented with AgNO_3_ and AgNPs keeping CuSO_4_ concentration as per standard of MS medium (0.025 mg/l) to study their effect on tissue culture responses of both genotypes (Table 3–A). In final step of study, the treatments/concentrations of CuNPs and AgNPs (optimum and below optimum) which demonstrated exceptional results on tissue culture responses of wheat were chosen and were applied in various combinations to study their effects on tissue culture responses of wheat genotypes.

#### 4.2.1. Plant Material and Sterilization

Mature caryopses of both genotypes were surface-sterilized with 90% ethanol for 5 min followed by thorough washing with four changes of sterile distilled water. The seeds were again sterilized for 25 min with 6.5% solution of sodium hypochlorite containing 0.1% Tween-20. Sterilized seeds were rinsed ﬁve to six times with autoclaved deionized distilled water and then imbibed aseptically in sterile water for 6 h at 33°C. Swollen mature embryos were aseptically excised from the caryopsis with the help of forceps and scalpel.

#### 4.2.2. Callus Induction

The basal MS medium^16^ was supplemented with 30 g/L sucrose as carbon source and 7g/l agar as gelling agent. For induction medium, the concentration of auxin was standardized and MS basal medium was supplemented with various concentrations of 2,4-D (0, 1.0, 1.5, 2.0, 2.5, 3.0, 3.5, 4.0, 4.5 and 5.0 mg/l). The pH of the media was adjusted to 5.8 and then autoclaved at 121°C for 20 minutes at 105 kPa. After autoclave, the media was cooled to room temperature for solidification. One hundred mature embryo explants per treatment per replication were cultured on induction medium supplemented with various concentration of 2,4-D facing scutellum side up. The cultures were incubated in total darkness at 25 ± 1°C and media was refreshed after two weeks. After four weeks of primary culture, the callus induction rate was recorded prior to transfer of primary calli to subculture medium. Same procedure was adopted to study effect of CuSO4, AgNO3 and their nano particles (Tables 2, 3 and 4). Callus induction frequency was recorded using following formula:

$$\begin{array}{rl} & callus\;Induction\;Frequency\left(\%\right)\\ & =\frac{No.\;of\;explantscultured}{No.\;of\;explants\;transformed\;into\;callus}\times 100 \end{array}$$

#### 4.2.3. Embryogenic Callus

Initially, induction medium and subculture medium were kept similar to obtain optimum concentration of 2,4-D for maximum embryogenic callus formation of both genotypes (AS-2002 and Wafaq-2001). The calli derived each from various concentrations of 2,4-D (0, 1.0, 1.5, 2.0, 2.5, 3.0, 3.5, 4.0, 4.5 and 5.0 mg/l) were sub-cultured on MS medium supplemented with respective concentration of 2,4-D under cool, white fluorescent light (10 µmol/m^2^/s, 22°C, 16-h photoperiod) for two weeks. Non-embryogenic (NE) calli were characterized by soft, loose, watery nature and cream to brownish color, whereas embryogenic calli (E) were characterized by more pale in color, smooth, nodular and compact texture. Frequency of embryogenic callus was recorded after three bi-weekly subcultures using following formula:

$$\begin{array}{rl} & callus\;Induction\;Frequency\left(\%\right)\\ & =\frac{No.\;of\;explants\;cultured}{No.\;of\;explants\;transformed\;into\;callus}\times 100 \end{array}$$

#### 4.2.4. Regeneration

The MS basal media was supplemented with diﬀerent combinations of Indole-3-acetic acid (IAA), 6-Benzylaminopurine (BAP) and Kinetin (Kin) to test their effect and to standardize regeneration medium for each genotype (Table-1). For each regeneration protocol, five hundred mature explants were cultured on standardized callus induction media offering maximum embryogenic calli in preceding study. One hundred uniform embryogenic calli were selected and incubated per treatment per replication of regeneration media. Same procedure was followed to study effects of CuSO_4_, AgNO_3_ and their nano particles (Tables 2, 3 & 4). After 4–5 weeks, regeneration frequency was recorded using formula given below:

$$\begin{array}{rl} & callus\;Induction\;Frequency\left(\%\right)\\ & =\frac{No.\;of\;explants\;cultured}{No.\;of\;explants\;transformed\;into\;callus}\times 100 \end{array}$$

#### 4.2.5. Root Strengthening and Transplant of Regenerated Plants

Plantlets regenerated from embryogenic calli were transferred onto half-strength MS medium (containing 20 g/l sucrose and 7 g/l agar) for root strengthening. For hardening, the plantlets were exposed to a high light density (80–90 µmol/m^2^/s, 25°C, 16-h photoperiod). Plantlets were grown to a height of 8 cm and those with well-developing roots were removed from the culture medium, washed gently under running water, transplanted to a mixture of vermiculite, perlite and moss (1:1:1) and grown (3–4 weeks) in the greenhouse to maturity.

### 4.3. Experimental Design

The experiments were laid out following Completely Randomized Design (CRD) with factorial arrangement, replicated thrice. The data collected were analyzed using Analysis of Variance (ANOVA) and treatments means were compared using Least Significant Difference (LSD) test (α = 0.05) using Statistics 8.1.1.0 software.

## Conclusion

Present study was aimed to study the effect of CuSO_4_, AgNO_3_ and their nanoparticles on tissue culture responses of mature embryo culture of wheat genotypes. In this study, the optimized induction medium fortified with various concentrations of CuSO_4_, CuNPs confirmed significant effect on frequency of embryogenic callus. Significantly higher regeneration can be achieved from MS-based regeneration medium supplemented with 0.015 mg/l or 0.020 mg/l CuNPs than standard 0.025 mg/l CuSO_4_. The maximum embryogenic calli are obtained from medium fortified with 3 mg/l or 4 mg/l AgNPs compared with control and rest of the treatments. The regeneration medium fortified with reduced concentration of Ag in the form of AgNPs (3 mg/l) or 5.0 mg/l as AgNO_3_ was at par and significantly improved regeneration of mature embryo explants of wheat genotypes compared with control. We also study individual and combined effect of Cu and Ag nanoparticles along with control (basal regeneration media of each genotype). Co-application of metallic NPs significantly increased embryogenic callus formation of genotypes. Induction medium supplemented with 0.015 mg/l CuNPs + 4 mg/l AgNPs or 0.020 mg/l CuNPs + 2 mg/l AgNPs showed splendid results than control and other combination of Cu and Ag nanoparticles. The maximum regeneration was achieved with combined application of 0.015 mg/l CuNP and 4 mg/l AgNPs with an increment of 21% in regeneration over control. It is revealed that CuNPs and AgNPs are potential candidate to augment somatic embryogenesis and regeneration of mature embryo explants of wheat. However, due to antimicrobial activity of Ag and Cu nanoparticles, their potential use in agrobacterium mediated genetic transformation needs to be addressed.

## Supplementary Material

Supplemental Material
