# Supplementary material for: Exploring potential of copper and silver nano particles to establish efficient callogenesis and regeneration system for wheat (Triticum aestivum L.)
Source: GM Crops Food. 2021 May 3;12(1):564–85. doi: 10.1080/21645698.2021.1917975 (PMC8820254; doi:10.1080/21645698.2021.1917975)
Supplement: Supplemental Material [file KGMC_A_1917975_SM2882.zip › Document.rtf]

Supplementary Materials: S1- All the combinations for optimization of callus induction and callus regeneration mediums with different growth regulators and Cu/Ag Nano-particles
